# Macromolecular crystallography at SPring-8 and SACLA

**DOI:** 10.1107/S1600577525000657

**Published:** 2025-02-18

**Authors:** Masaki Yamamoto, Takashi Kumasaka

**Affiliations:** a RIKEN SPring-8 Center, 1-1-1 Kouto, Sayo, Hyogo679-5148, Japan; b Japan Synchrotron Radiation Research Institute (JASRI) 1-1-1 Kouto Sayo Hyogo679-5198 Japan; University of Manchester, United Kingdom

**Keywords:** synchrotron radiation, X-rayfree electron lasers, XFELs, SPring-8, SACLA, macromolecular crystallography

## Abstract

Since the groundbreaking determination of the first protein crystal structure by J. C. Kendrew in 1959, macromolecular crystallography (MX) has remained at the forefront of structural biology, driven by continuous technological advancements. The advent of synchrotron radiation in the 1990s revolutionized the field, enhancing data quality, introducing novel phasing methods, and broadening the scope of target samples to include membrane proteins and supramolecular complexes. In 1997, Japan inaugurated SPring-8, one of the world’s largest third-generation synchrotron radiation facilities. With its high-brilliance radiation from insertion devices, SPring-8 has dramatically increased the capability of MX. This paper describes MX’s evolution, current developments, and prospects at SPring-8 and SACLA.

## Abstract 2

This paper presents the development of instrumentation and macromolecular crystallography methods at the third-generation synchrotron facility SPring-8 and X-ray free electron laser facility SACLA. The aim is to facilitate rapid structural analysis from highly challenging protein crystals.

## 1. Synchrotron radiation in macromolecular crystallography

The 21st century is heralded as the ‘era of life science’, characterized by groundbreaking discoveries with profound implications across medicine, biotechnology and industry. Central to this progress is the study of proteins, which, guided by genetic information, assume intricate three-dimensional structures essential for biological functions. X-ray crystallography has long been established as the premier analytical method for elucidating protein structures (Blundell & Johnson, 1976 ▸).

Synchrotron radiation (SR) has emerged as an indispensable tool for structural biology, providing unparalleled advantages in studying proteins (Helliwell, 1992 ▸; Smith *et al.*, 2012 ▸; Owen *et al.*, 2016 ▸; Yamamoto *et al.*, 2017 ▸; Hendrickson, 2000 ▸). The pioneering work of G. Rosenbaum, K. C. Holmes and colleagues in 1971 marked the advent of SR-based biological structural studies. Their experiments at the electron synchrotron DESY in Germany produced the first fibre diffraction images of muscle (Rosenbaum *et al.*, 1971 ▸). Shortly after, K. O. Hodgson and collaborators captured the first diffraction photographs from protein crystals at the synchrotron SPEAR, Stanford University (Phillips *et al.*, 1976 ▸). These studies revealed SR’s distinct advantages, including beam intensities 50–60 times greater than laboratory X-ray sources and tuneable wavelengths that enable anomalous dispersion, establishing SR as a transformative tool for macromolecular crystallography (MX).

These initial successes catalysed the development of dedicated MX beamlines at second-generation SR facilities such as the Daresbury SRS in the UK (Helliwell *et al.*, 1982 ▸), NSLS at Brookhaven National Laboratory in the USA (Berman *et al.*, 1992 ▸) and the Photon Factory (PF) at the Institute of High Energy Physics in Japan during the 1980s (Sakabe, 1983 ▸). SR applications in MX flourished, driven by continual advancements in experimental techniques. In 1985, J. Deisenhofer, R. Huber and H. Michel successfully determined the first crystal structure of a membrane protein, the photosynthetic reaction centre, an achievement recognized with the 1988 Nobel Prize in Chemistry (Deisenhofer *et al.*, 1985 ▸; The Nobel Prize in Chemistry, 1988 ▸). Despite these advancements, structural analysis remained challenging, with the number of deposited protein structures in databases a mere fraction of today’s figures. Membrane protein studies, in particular, were exceedingly rare, with notable exceptions such as the 1995 determination of cytochrome c oxidase by T. Tsukihara *et al.* in Japan (Tsukihara *et al.*, 1996 ▸).

The 1990s witnessed third-generation SR facilities constructed with insertion devices to produce highly brilliant light sources. Facilities such as the ESRF in Europe (Laclare, 1994 ▸), the APS in the USA (Moncton, 1998 ▸) and SPring-8 in Japan (Kamitsubo, 1998 ▸) have dramatically increased the capabilities of MX. These high-brilliance beamlines, coupled with advances in molecular biology techniques for protein expression and purification, enabled the structural analysis of increasingly complex macromolecules, including large and membrane proteins. On the other hand, the second-generation SR facility of KEK/PF has been developing native-SAD (native single-wavelength anomalous dispersion) using sulfur atoms (Liebschner *et al.*, 2016 ▸; Basu *et al.*, 2019 ▸), which are naturally present in protein, and is still being used as a complement to SPring-8, which specializes in high-brilliance microbeams.

As structural biology emerged in the post-genome era, the field focused on deciphering the relationship between structure and function in proteins and macromolecules that govern biological processes. Since its inception in 1997, SPring-8 has been at the forefront of structural biology research, with a particular emphasis on MX. Through continuous innovation, SPring-8 has contributed significantly to advancing structural biology, enabling new insights into the molecular mechanisms underlying life.

## 2. Progress in MX at SPring-8

SR has played a transformative role in MX, with its key contributions summarized as achieving higher accuracy in structural analysis, expanding the diversity of analysable sample targets, and enhancing the speed and simplicity of the analysis process.

**Figure 1:**
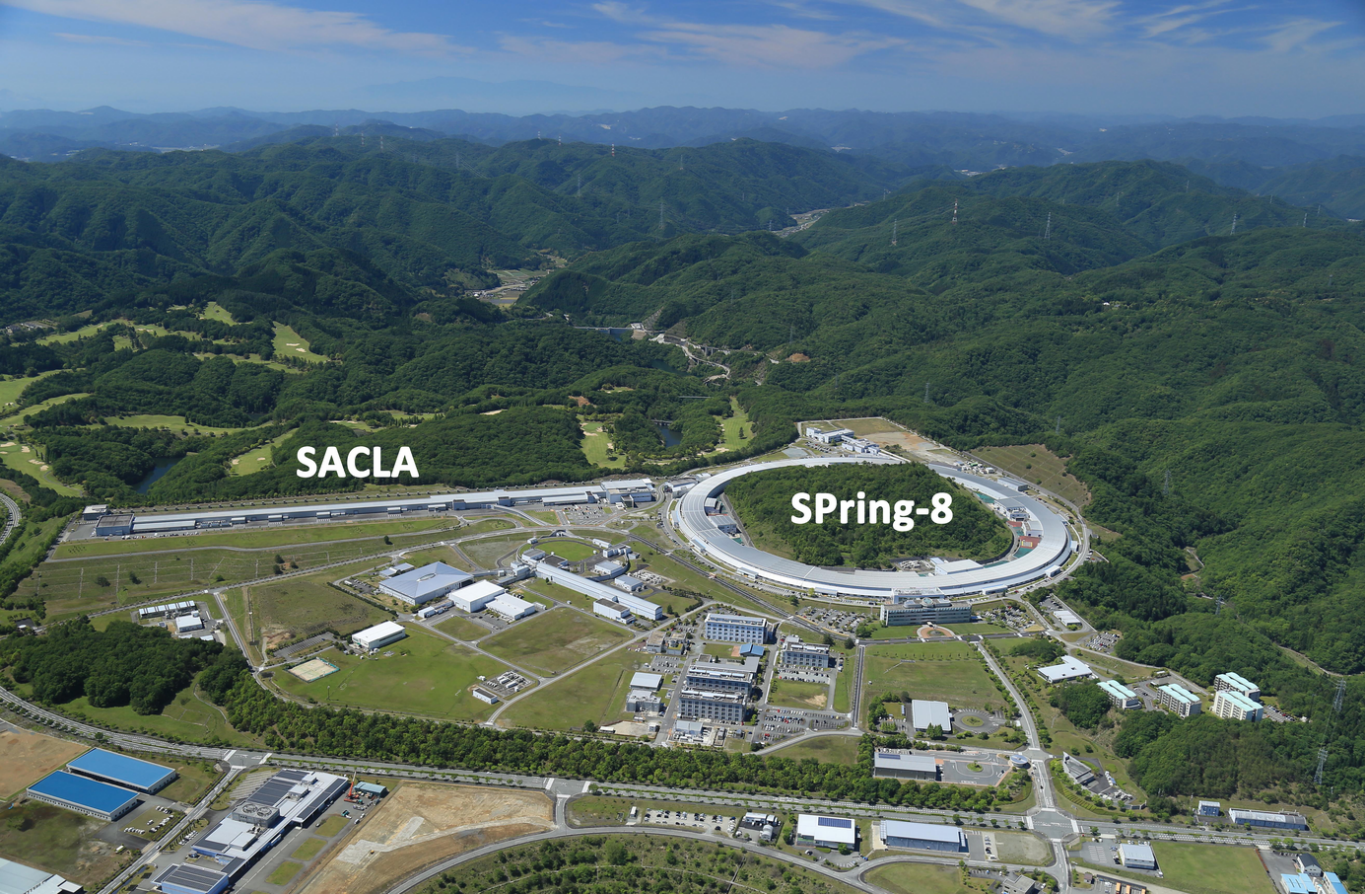
Overall view of SPring-8 and SACLA – SPring-8 with a circumference of 1436 m surrounding Mihara Kuriyama and a straight-line 700 m-long SACLA at the SPring-8 campus.

In 1997, SPring-8, the last large-scale third-generation SR facility, was constructed by a collaborative effort between RIKEN and JAERI (Kamitsubo, 1998 ▸) (Fig. 1 ▸). Among its initial advancements was the establishment of two specialized undulator beamlines, BL41XU (Kamiya *et al.*, 1995 ▸) and BL45XU (Yamamoto *et al.*, 1998 ▸), designed to enable high-accuracy data collection for MX. Around this time, low-temperature cryogenic diffraction experiments (Hope, 1988 ▸; Rodgers, 1994 ▸; Teng & Moffat, 2002 ▸) became widespread to mitigate radiation damage caused by high-intensity X-rays (Garman, 1999 ▸; Walsh *et al.*, 1999 ▸), paving the way for high-resolution analysis of weakly diffracting protein crystals.

**Figure 2:**
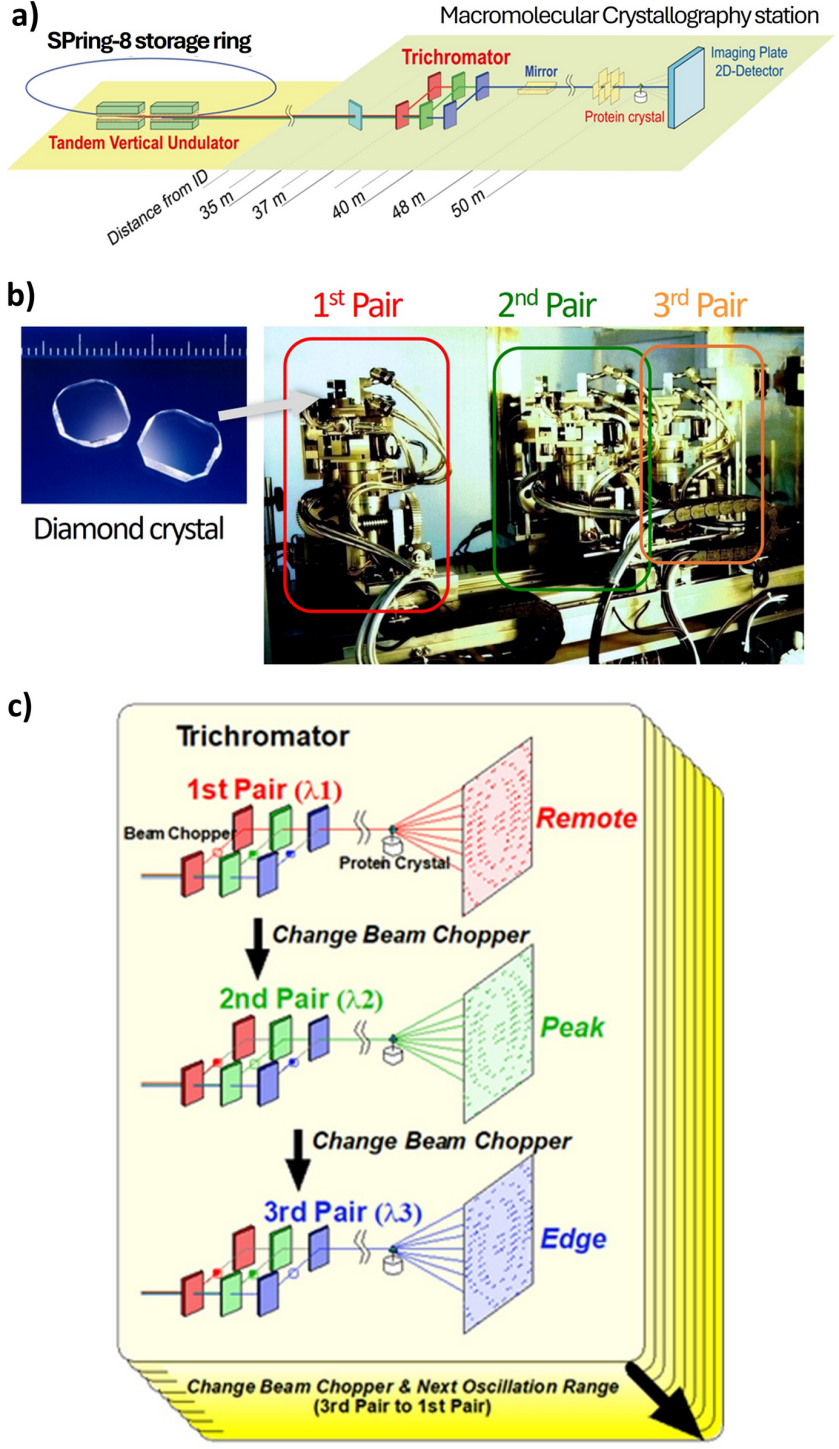
Trichromatic concept at BL45XU. (*a*) Diagram of the main components of BL45XU, (*b*) trichromator consisting of three pairs of double-crystal synthetic diamonds, and (*c*) the trichromatic concept for measuring high-precision MAD data while rapidly switching between three wavelengths by trichromator.

The tuneable wavelength capability of SR revolutionized phase determination in MX. By leveraging changes in diffraction intensity caused by anomalous dispersion effects, the multiple-wavelength anomalous dispersion (MAD) method enabled precise phase determination using a single protein crystal with anomalously scattering atoms (*e.g.* metals) (Hendrickson, 1991 ▸). At SPring-8, the early years of BL45XU saw the development of the innovative trichromator, which employs three fixed-exit double-crystal diamond monochromators. Diamond, with its low atomic number, minimized X-ray absorption and allowed the transmission of X-rays that were not aligned with monochromatizing Bragg conditions. This design enabled the simultaneous production of three monochromatic X-ray wavelengths on a single beam path, facilitating the near-simultaneous collection of anomalous diffraction data without altering the trichromator settings – a strategy termed the ‘trichromatic concept’ (Fig. 2 ▸) (Yamamoto *et al.*, 1995 ▸; Kumasaka *et al.*, 2002 ▸).

Integrating this trichromatic approach with cryogenic diffraction techniques allowed SPring-8 to solve structures of protein crystals deemed intractable at other facilities. Notable successes include the determination of challenging protein structures, such as rhodopsin (Palczewski *et al.*, 2000 ▸), glutamate receptor (Kunishima *et al.*, 2000 ▸) and flagellar proteins (Samatey *et al.*, 2001 ▸). Over time, the continuous construction and refinement of MX beamlines at SPring-8 culminated in the establishment of six operational MX beamlines, each tailored for specific structural biology applications.

The advances in beamline performance and improvements in detector technology and phase determination software have elevated the single-wavelength anomalous dispersion (SAD) method to the forefront of experimental phase determination (Rice *et al.*, 2000 ▸). This streamlined approach has significantly reduced the complexity of data collection and analysis.

Among the landmark achievements at SPring-8 are the structural determination of Ca-ATPase by Toyoshima *et al.* (2000 ▸) and the groundbreaking resolution of the first crystallographic structure of a G-protein coupled receptor (GPCR), bovine rhodopsin, by Palczewski *et al.* (2000 ▸). These milestones underscore SPring-8’s pivotal role in advancing the frontier of structural biology and its continued leadership in MX research.

### 2.1. Ultra-high-resolution structural analysis using high-energy X-rays

The high brightness and flux of SR have significantly advanced MX’s measurement accuracy and analytical capabilities. These features are indispensable for resolving the minute diffraction signals produced by protein crystals, primarily consisting of light atoms like carbon, nitro­gen, oxygen and hydrogen with low X-ray scattering cross sections. The improved signal-to-noise ratio (S/N) of weak diffraction spots in high-resolution regions – once undetectable due to low diffraction intensity – has enabled structural analyses to approach atomic resolution. Utilizing high-energy X-rays across SR’s broad energy spectrum further facilitates discussions of chemical structures at resolutions beyond 1.0 Å.

**Figure 3:**
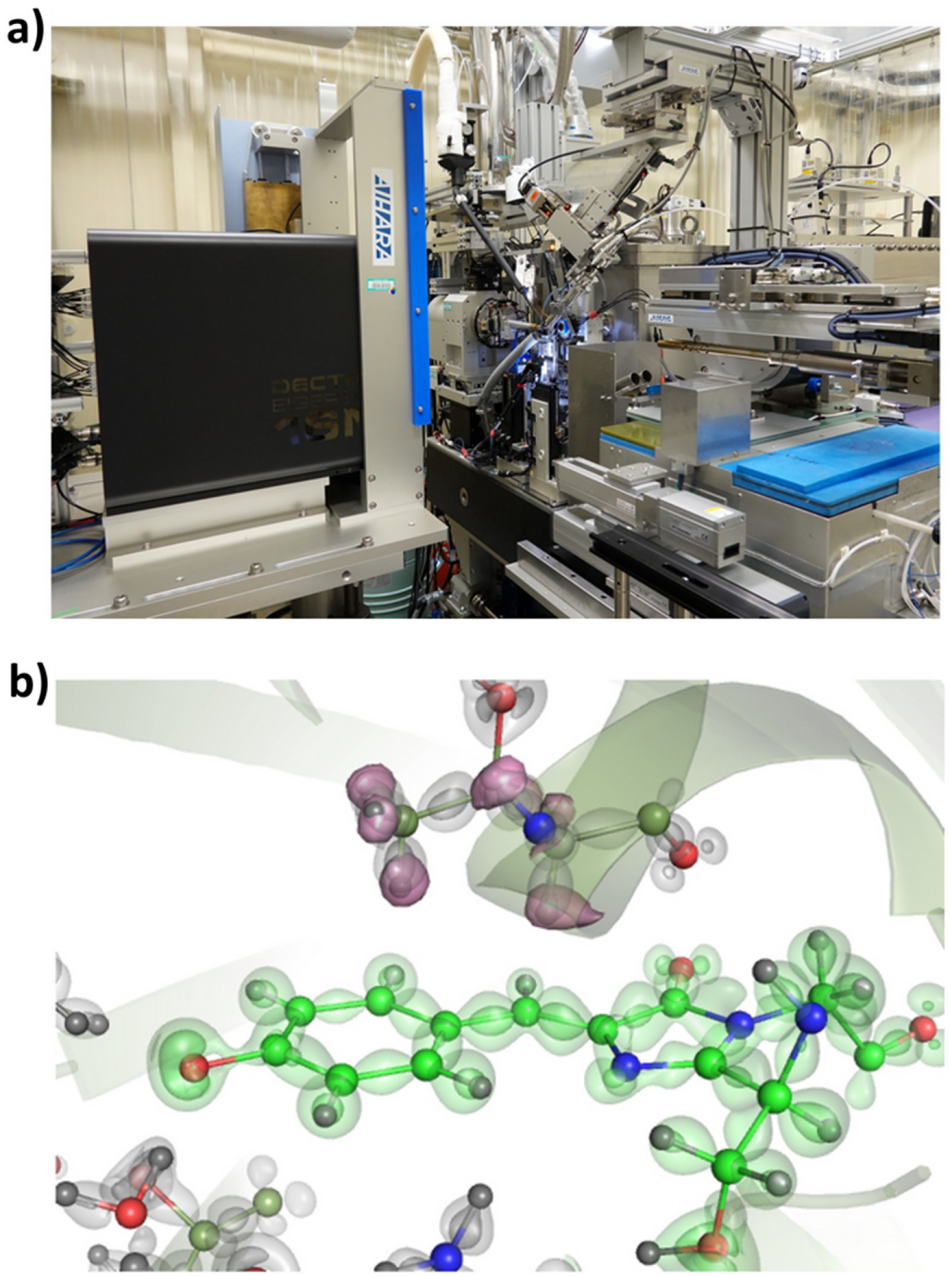
(*a*) Experimental station of BL41XU and (*b*) electron density at 0.79 Å of GFP chromogenic centres. Static deformation maps, the difference between the multipolar and the normal spherical atomic models, are shown for the chromophore and the surrounding residues in green and grey surfaces, respectively.

At SPring-8’s BL41XU beamline (Hasegawa *et al.*, 2013 ▸) [Fig. 3 ▸(*a*)], diffraction experiments employ high-energy X-rays with wavelengths ranging from 0.35 to 0.60 Å in addition to the conventional 1.0 Å. A dedicated high-energy diffractometer is installed in an upstream experimental hutch of the beamline, enabling precise determination of elemental positions based on their absorption edges and the collection of ultra-high-resolution diffraction data. In a remarkable achievement, the world’s highest resolution of 0.48 Å was obtained by K. Miki’s group from Kyoto University. Their study on high-potential iron–sulfur proteins (HiPIPs) visualized the electron densities of 3*d* electrons on iron atoms and 3*p* electrons around sulfur atoms, demonstrating the power of ultra-high-resolution structural analysis (Hirano *et al.*, 2016 ▸). Structure determination at ultra-high resolution (>0.7 Å resolution) is expected to enable direct observation of the density distribution and orbitals of hydrogen atoms and outer-shell electrons, which play an essential role in the functional expression of proteins.

However, high-energy X-rays interact weakly with matter, and the low sensitivity of conventional X-ray detectors to high-energy photons makes collecting high-precision data challenging. This limitation arises from the weak diffraction intensity of samples and the insufficient detection efficiency of standard detectors. Since the mid-2010s, the introduction of two-dimensional detectors using CdTe (cadmium telluride) as the X-ray sensor – known for its high sensitivity to high-energy X-rays – has revolutionized the experimental environment.

A notable breakthrough was achieved through collaboration between K. Takeda and K. Miki’s group at Kyoto University and K. Hasegawa at the Japan Synchrotron Radiation Research Institute (JASRI). They conducted ultra-high-resolution diffraction experiments on green fluorescent protein (GFP) at BL41XU, testing the performance of the PILATUS3 X CdTe pixel array detector. Their experiments yielded data with a resolution of 0.78 Å at an absorption dose as low as 0.1 MGy. They successfully performed charge density analysis using a multipolar atomic model (Takaba *et al.*, 2019 ▸) [Fig. 3 ▸(*b*)].

Following the result, we installed the EIGER2 CdTe 4M detector at BL41XU in 2021. This state-of-the-art detector features CdTe sensors with a detection efficiency that is an order of magnitude higher in the high-energy range compared with conventional detectors. Its superior performance is expected to standardize high-energy X-ray diffraction experiments, offering new opportunities to visualize atomic and electronic structures with unparalleled precision. Notably, this technology provides a significant advantage to ultra-high-resolution studies of radiation-sensitive crystals (Fukuda *et al.*, 2024 ▸).

### 2.2. Generalization of MX and expansion of analysis targets

The expansion of analytical targets in MX has focused on challenging samples such as membrane proteins and large, complex structures. High-precision diffraction data collection, supported by advancements in membrane protein production, purification and crystallization technologies, alongside preparation methods for complex assemblies, has been made possible by SR. The high-resolution and high-precision structural analyses enabled by SR have significantly enhanced the accuracy of structural information, facilitating breakthroughs in understanding complex biological systems (Hendrickson, 2016 ▸).

A pivotal development in accelerating and simplifying structural analysis has been the adoption of phase determination methods such as MAD and SAD, mainly using seleno­methio­nine (Hendrickson, 1999 ▸; Deacon & Ealick, 1999 ▸; Terwilliger & Berendzen, 1999 ▸). The development of automated tools, such as the SPACE sample changer for frozen crystals (Ueno *et al.*, 2004 ▸), has dramatically increased the speed and efficiency of diffraction data collection, especially in structural genomics research. Innovations in sample changers (Ueno *et al.*, 2004 ▸; Murakami *et al.*, 2012 ▸, Murakami *et al.*, 2020 ▸) and remote and automatic beamline control software systems like *BSS* (Ueno *et al.*, 2005 ▸) have further enhanced measurement efficiency. These advancements have transformed MX into a more generalized and accessible technique, contributing to comprehensive protein structure analyses and also significantly advancing the field of structural biology.

By 2005, the combination of high-brilliance SR beamlines with MAD/SAD methods, low-temperature diffraction experiments, precision goniometers, CCD detectors (pixel array detector afterwards) and advanced data collection software established a robust framework for high-quality structural analysis. These developments enabled structural analyses from smaller crystals and higher resolutions, allowing the construction of atomic structural models.

**Figure 4:**
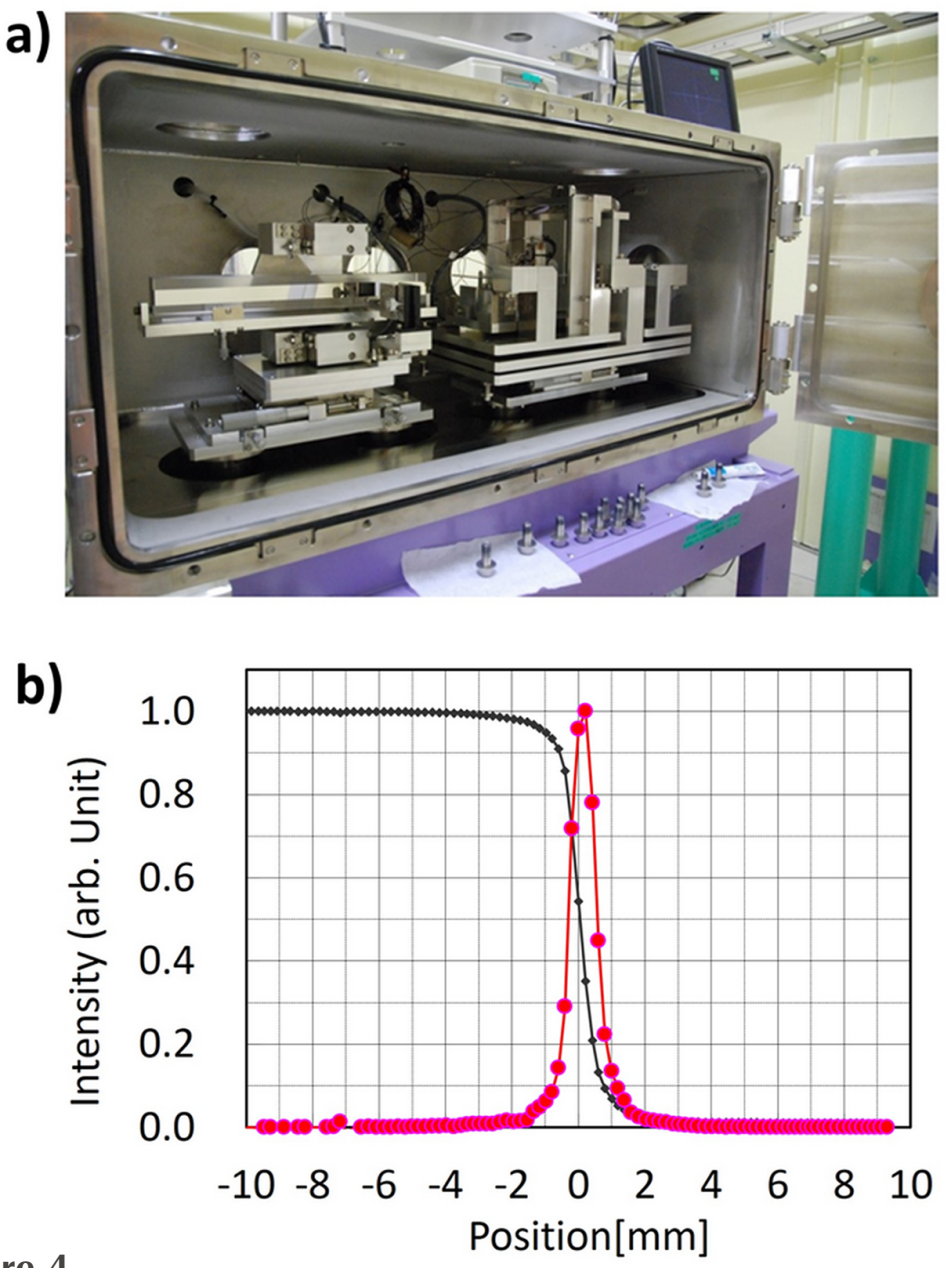
(*a*) Microbeam focusing optics (a two-dimensional focusing mirror polished by EEM) for BL32XU and (*b*) beam profile of an edge scan of a 1 µm beam.

These technological advancements culminated in the construction of the BL32XU beamline at SPring-8 (Hirata *et al.*, 2010 ▸). Operational since 2009, BL32XU delivers a high-flux microbeam of 6 × 10^10^ photons s^−1^ with a 1 µm focal spot, achieved using a two-dimensional focusing mirror polished by the elastic emission machining (EEM) method (Yamauchi *et al.*, 2003 ▸) (Fig. 4 ▸). This unparalleled capability has facilitated the structural analysis of microcrystals and previously intractable and highly challenging proteins.

Building on the successes of BL32XU, the optical system of BL41XU was upgraded in 2014 (Hasegawa *et al.*, 2013 ▸), further improving its performance. In 2019, the small-angle X-ray scattering (SAXS) beamline at BL45XU was reconfigured into an MX beamline, enabling data collection with a minimum microbeam size of 5 µm (Goto *et al.*, 2019 ▸). These enhancements have continued to expand the applicability of MX and enable cutting-edge structural analyses of even the most complex biological systems.

### 2.3. Microcrystallography and high-throughput analysis

The lipidic cubic phase (LCP) method, introduced by Landau & Rosenbusch (1996) ▸, revolutionized the structural analysis of membrane proteins, key targets for understanding biological processes and advancing drug discovery (Lima *et al.*, 2020 ▸; Douangamath *et al.*, 2021 ▸). By reconstituting membrane proteins solubilized with surfactants into a three-dimensional lipid bilayer for crystallization, the LCP method mimics the natural environment of cell membranes. However, obtaining crystals large enough (tens of micrometres) for conventional crystallographic analysis remains challenging, despite the innovation.

The BL32XU beamline at SPring-8 has been at the forefront of addressing the limitations of microcrystal analysis. An automated data acquisition system, ‘*ZOO*’, was developed to facilitate structural determination from numerous protein microcrystals (Hirata *et al.*, 2019 ▸). *ZOO* integrates several automated tools:

(i) *SHIKA*. Identifies crystal positions through high-speed two-dimensional scanning of microcrystals that are invisible to the naked eye.

(ii) *KUMA*. Collects high-quality data while minimizing radiation damage to sample crystals.

(iii) *KAMO*. Processes diffraction data for structural analysis almost automatically (Yamashita *et al.*, 2018 ▸).

The *ZOO* system is also seamlessly linked to the SPACE sample changer, enabling fully automated diffraction intensity data collection across various measurement schemes, including: (*a*) single-crystal data collection, (*b*) helical data collection (Flot *et al.*, 2010 ▸), (*c*) multiple partial data accumulation (small-wedge synchrotron crystallography, SWSX), (*d*) mixed schemes (*e.g.* helical data collection combined with SWSX), (*e*) serial synchrotron rotation crystallography (SS-ROX) (Gati *et al.*, 2014 ▸; Hasegawa *et al.*, 2017 ▸, 2021 ▸).

**Figure 5:**
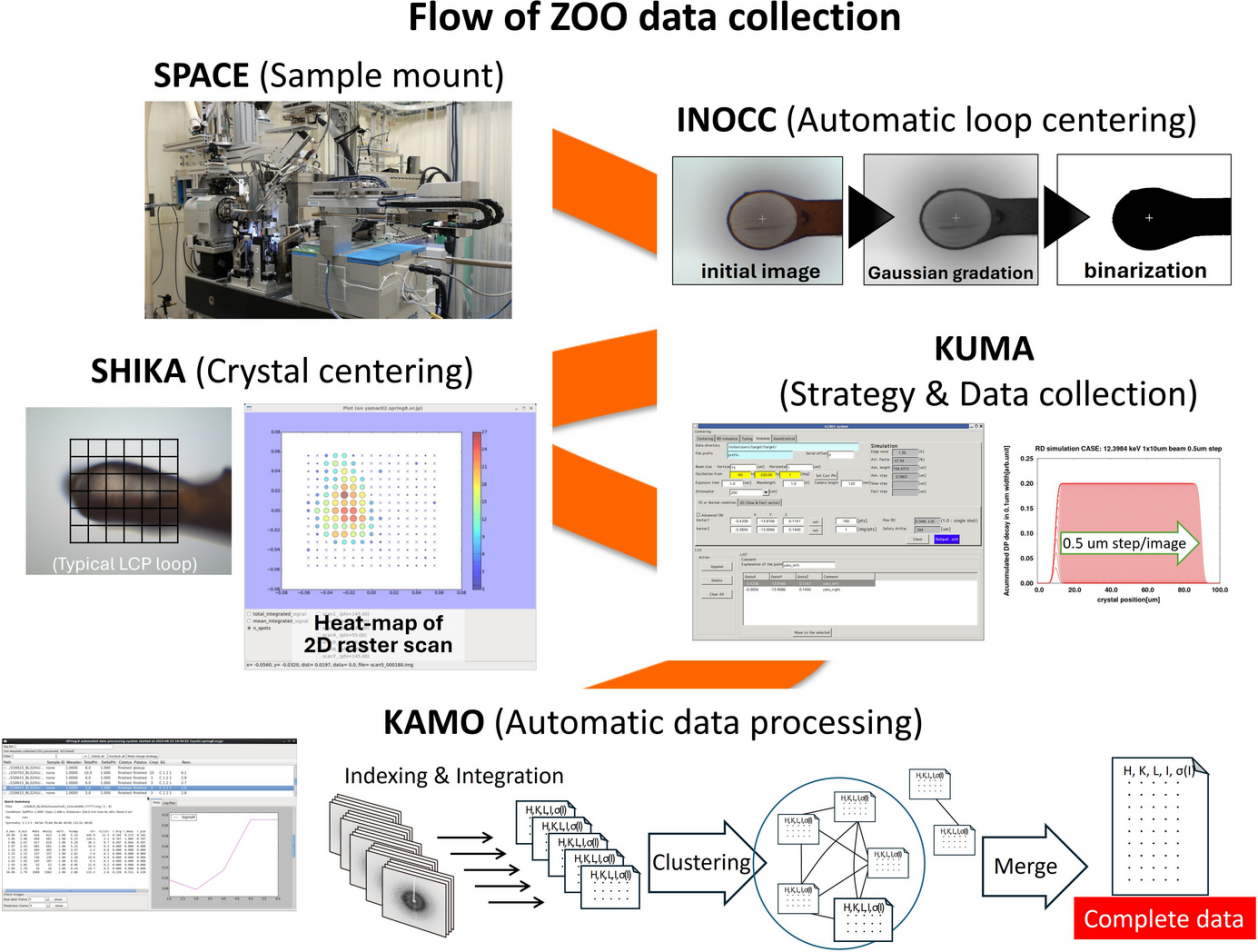
Flow of automatic diffraction intensity data collection in the *ZOO* system.

**Figure 6:**
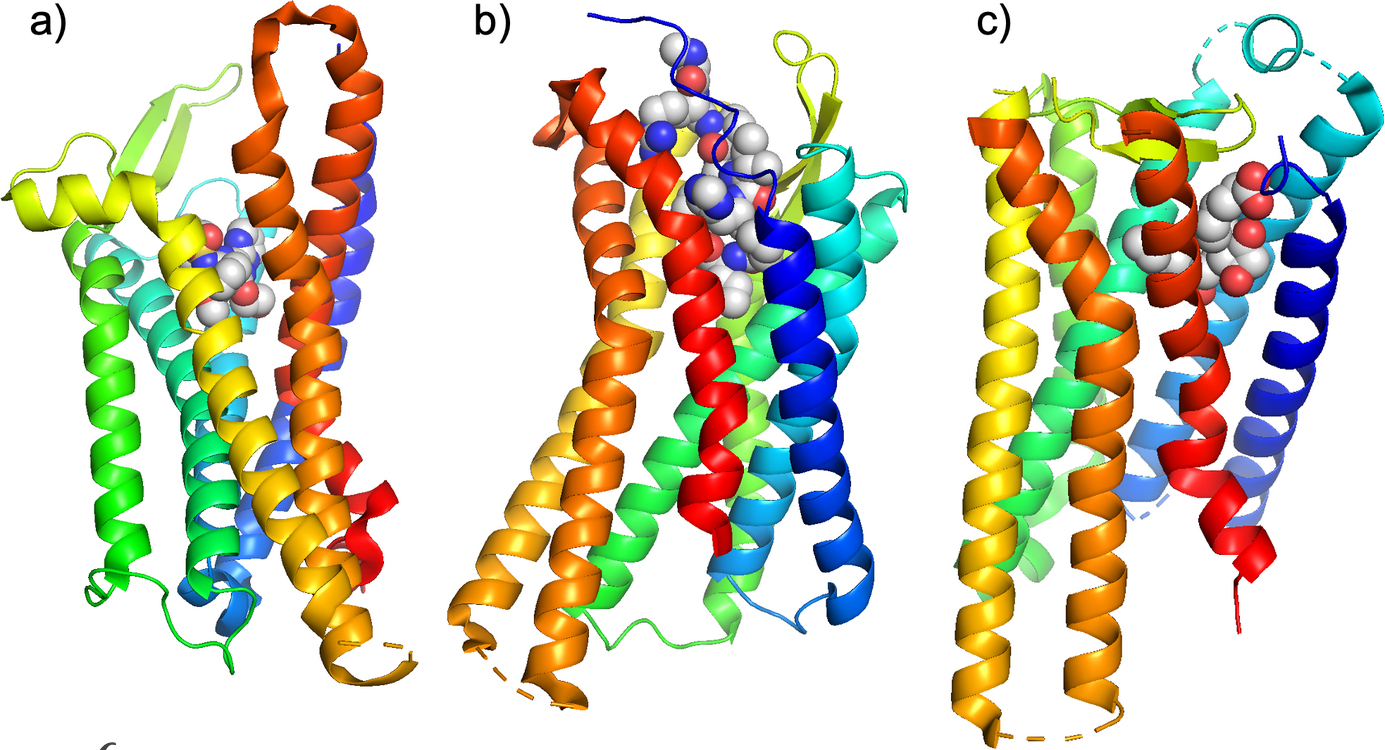
Examples of GPCR structures solved using the *ZOO* system. (*a*) Endothelin receptor (PDB: 5xpr), (*b*) angiotensin II receptor (PDB: 5xjm), (*c*) prostaglandin E receptor (PDB: 6ak3).

Fig. 5 ▸ illustrates the automatic diffraction intensity data collection flow in *ZOO*, while Fig. 6 ▸ provides examples of successful applications.

A significant innovation enabled by *ZOO* is high-data-rate macromolecular crystallography (HDR-MX), which involves collecting extensive diffraction data from many crystals at high data rates. This approach has made high-resolution crystallographic analysis more accessible to researchers without requiring specialized expertise (Bernstein *et al.*, 2020 ▸).

**Figure 7:**
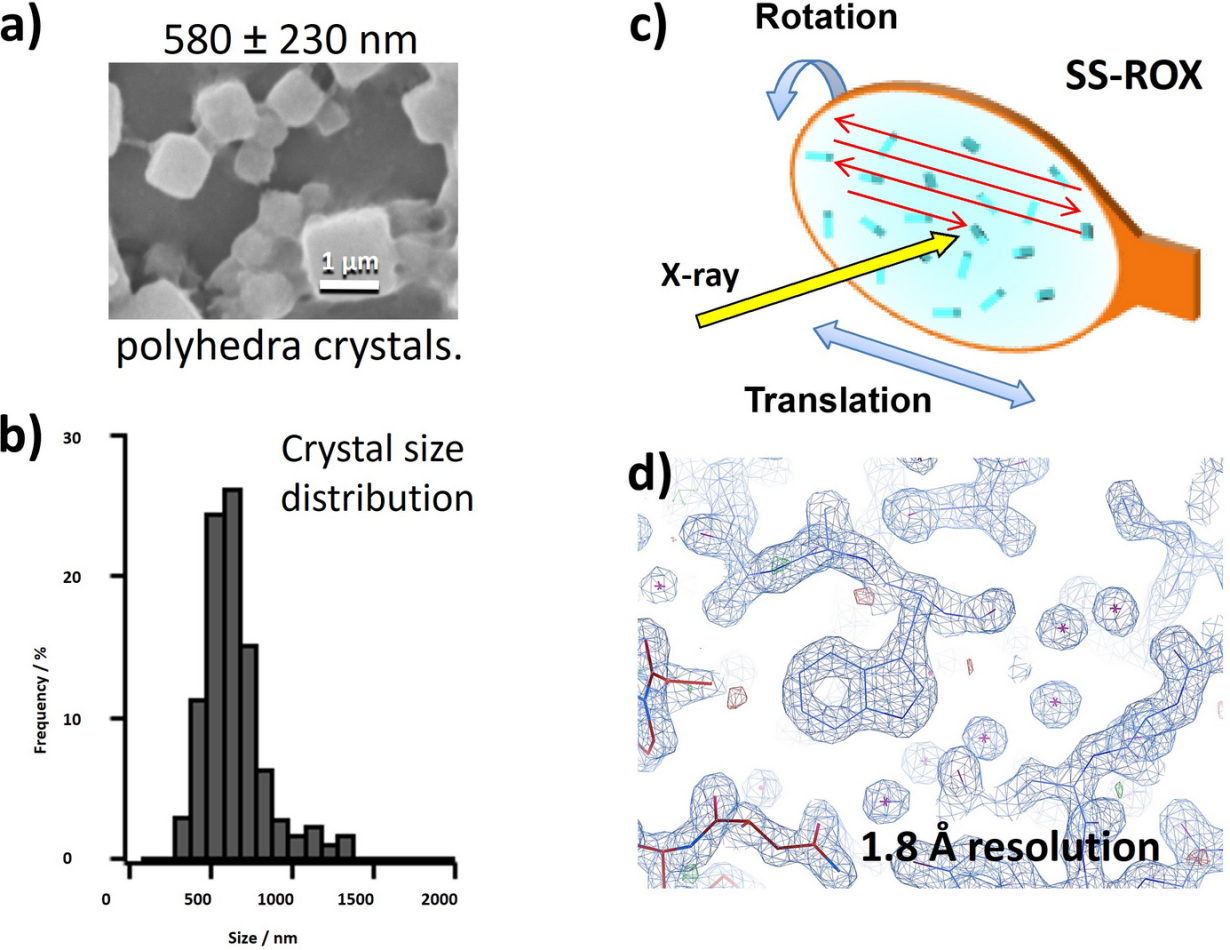
High-resolution structural analysis from submicrometre crystals by HDR-MX with the *ZOO* system. (*a*) Submicrometre crystal and (*b*) crystal size distribution. (*c*) Conceptual diagram of SS-ROX method measurement and (*d*) electron-density diagram analysed from a polygonal submicrometre crystal. A high-resolution structure at 1.8 Å resolution was successfully obtained by SS-ROX from submicrometre crystals of ‘polyhedra’ of several hundred nm in size, which crystallize autonomously in cells by cell-free protein synthesis.

HDR-MX has transformed MX by enabling the efficient collection of diffraction data from many crystals, addressing challenges posed by tiny crystals with weak diffraction intensities that were previously unmeasurable. A remarkable achievement of HDR-MX was led by T. Ueno’s group at the Tokyo Institute of Technology in collaboration with K. Hirata at RIKEN. Using the high-brilliance microbeam at BL32XU and SS-ROX data collection via *ZOO*, they successfully analysed sub-micrometre crystals (580 nm in size). They resolved their structure at 1.8 Å resolution (shown in Fig. 7 ▸) (Abe *et al.*, 2022 ▸). This milestone represents high-resolution structural analysis from ultra-small crystals, comprising crystal lattices of 10^5^ orders – far smaller than the 10^8^–10^9^ lattices once considered necessary for high-resolution X-ray imaging (Moukhametzianov *et al.*, 2008 ▸).

HDR-MX exemplifies how innovations in MX beamlines, including automated data acquisition and high-brilliance microbeams, have expanded the scope of structural biology. Moving forward, the generalization of HDR-MX is expected to enable rapid, high-resolution structural analyses of challenging proteins from sub-microcrystals, further advancing the field of MX.

## 3. Use of X-ray free electron lasers for structure–function research

The X-ray free electron laser (XFEL) represents a groundbreaking advancement in light-source technology, first introduced at the Linac Coherent Light Source (LCLS) in the USA and subsequently developed at the SPring-8 Angstrom Compact Laser (SACLA) in Japan (Ishikawa *et al.*, 2012 ▸). XFEL is a low-repetition, high-intensity, coherent pulsed X-ray with an extremely short femtosecond pulse width. Unlike SR, which is continuous at a constant intensity, XFEL can record snapshots of the transient state of the sample in a very short time. XFEL enables the precise observation of protein reaction processes and other *in vivo* phenomena in their native ‘*in situ*’ state on a femtosecond timescale, overcoming the limitations of SR in temporal resolution of sub-milliseconds and radiation damage. This section discusses the application of XFEL in damage-free X-ray crystallography and high-resolution time-resolved structural analysis, both of which capitalize on the XFEL’s femtosecond-scale high-intensity pulsed X-rays to capture snapshots of dynamic processes.

At the heart of the XFEL’s capabilities lies the principle of ‘diffraction before destruction’ (Neutze *et al.*, 2000 ▸). When high-intensity XFEL light irradiates a sample, ionization-induced destruction occurs within tens of picoseconds. However, the diffraction image is captured within the ultrashort femtosecond pulse duration before the onset of significant radiation damage. This allows for damage-free structural analysis, even of sensitive samples, and has enabled significant innovations in crystallographic methods.

**Figure 8:**
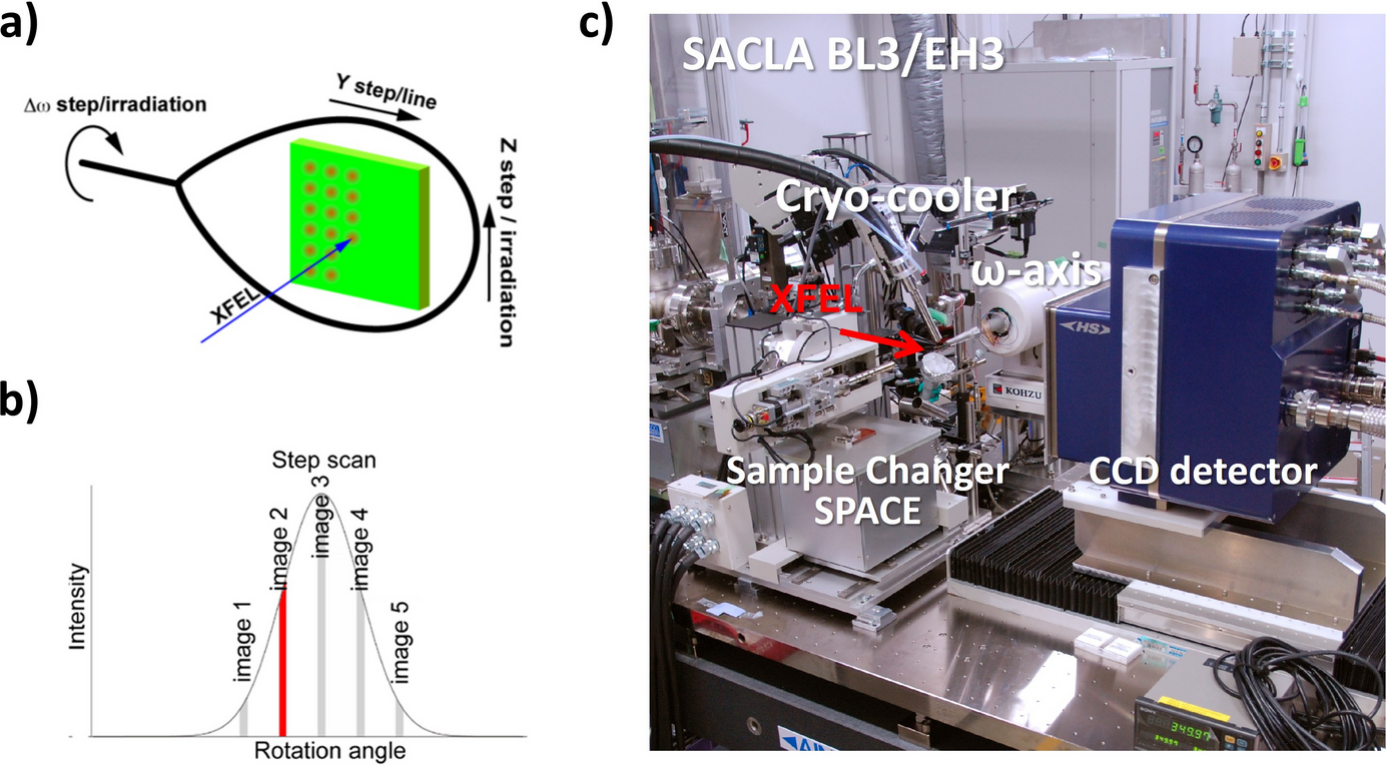
Schematic of serial femtosecond rotation crystallography (SF-ROX). (*a*) The data collection approach of SF-ROX rotated a large single crystal with a size range of several hundred micrometres by a small angle in a stepwise fashion to record still diffraction images that are discrete and sequential during the crystal rotation. (*b*) SF-ROX simplifies and improves the diffraction intensity integration at the XFEL by sampling the diffraction profiles in continuous still images. (*c*) Experimental setup of SF-ROX at SACLA.

One such innovation is ‘serial femtosecond rotation crystallography (SF-ROX)’, developed at SACLA. In SF-ROX, high-resolution diffraction images are collected from large crystals by systematically varying the irradiation position for each XFEL pulse using a general diffractometer integrated into the XFEL system. The larger crystal volume enhances diffraction signal quality, facilitating high-resolution analysis (Fig. 8 ▸). This approach has been applied to study radiation-sensitive samples, such as the oxygen reduction reaction in cytochrome oxidase. SACLA researchers successfully resolved the undamaged structure of the active centre involved in oxygen reduction at a resolution of 1.9 Å (Hirata *et al.*, 2014 ▸).

Another pivotal XFEL method is serial femtosecond crystallography (SFX), which involves introducing microcrystals into the XFEL irradiation field using a liquid beam or similar delivery systems. Diffraction data are then collected for each XFEL pulse. Initially demonstrated at LCLS, SFX has produced numerous insights into micrometre-sized crystals (Chapman *et al.*, 2011 ▸). At SACLA, extensive development of the SFX system has been undertaken, broadening its application to various challenging protein structures. One of the outstanding results is the study of the photoactivation mechanism of bacteriorhodopsin (Nango *et al.*, 2016 ▸).

Looking ahead, XFEL research in protein structure should continue to refine damage-free static structural analysis techniques while advancing dynamic structure analysis methods. Dynamic analysis aims to resolve structural changes during protein function, including short-lived reaction intermediates, by controlling reaction processes with external pump light (Aquila *et al.*, 2012 ▸). Such analyses are key to elucidating protein reaction mechanisms and physiological functions at atomic and electronic levels. These advancements in XFEL-based research are expected to offer critical insights into reaction chemistry, paving the way for a deeper understanding of biological functions.

## 4. Structural dynamics study at SPring-8

### 4.1. Structural polymorphism analysis by HDR-MX

The structural analysis approach discussed in Section 2.3, which uses the SWSX method and frozen crystals to merge large volumes of diffraction data, represents a significant development in crystallography. At SPring-8, this methodology is being further developed to enable dynamic structural analysis on the millisecond timescale as a complement to XFEL studies.

One challenge in HDR-MX using the *ZOO* system is the potential need for isomorphism in the crystal lattice and statistical inconsistencies in diffraction intensity data. Traditional assumptions – that all protein molecules within a crystal are structurally identical under the same crystallization conditions – often overlook structural fluctuations or heterogeneous conformations within the asymmetric units or across reaction states.

To address this, SPring-8 employs hierarchical clustering through *KAMO* to analyse the extensive diffraction datasets. By grouping diffraction patterns indicative of structural diversity, the system can independently merge and analyse data from each group. An automated pipeline, ‘NABE’, further streamlines structural analysis by listing and visualizing results (Matsuura *et al.*, 2023 ▸).

**Figure 9:**
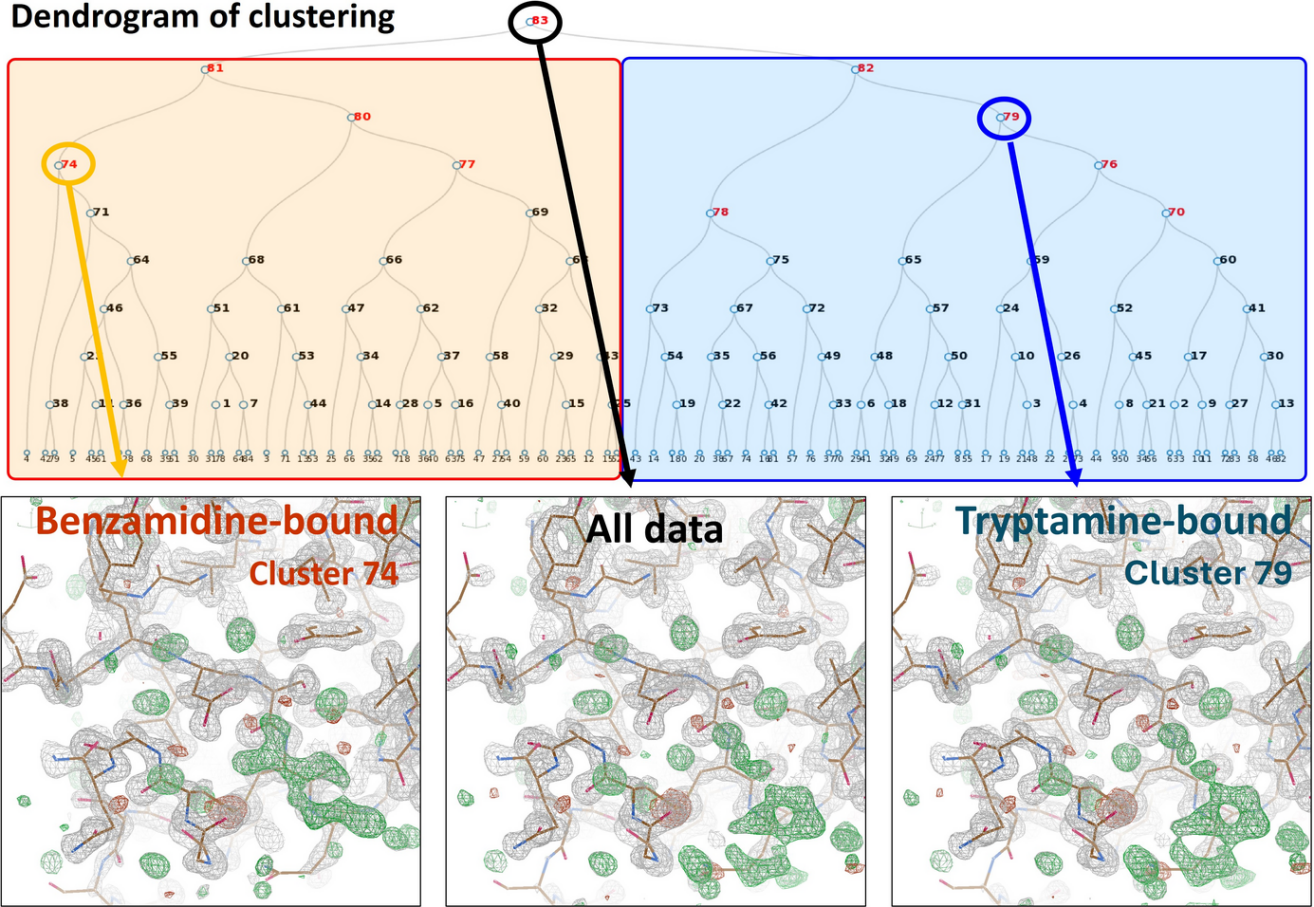
Hierarchical clustering of two different trypsin compound complex datasets. Dendrogram of diffraction data containing two compound trypsin complexes classified by hierarchical clustering based on intensity correlation by *KAMO* and electron density of compound binding sites analysed from different clusters by NABE.

For example, hierarchical clustering was applied to a mixed dataset containing diffraction data from two distinct compound complexes of trypsin, one bound to benzamidine and the other to tryptamine. The dataset was classified using NABE, and molecular replacement and refinement were performed for each cluster. Electron densities for compound binding sites, previously unclear in the topmost cluster, became distinguishable in sub-clusters corresponding to each compound’s unique binding structure (Fig. 9 ▸).

This approach has broad implications for understanding proteins with biochemically meaningful structural heterogeneity within asymmetric units, enabling more profound insights into mechanisms of action. By combining microbeam data collection with hierarchical clustering, researchers can classify and extract such structural variations, offering significant potential for future time-resolved structural studies. Automating data measurement has enabled an order-of-magnitude increase in data collection, further enhancing structural analysis capabilities.

### 4.2. Ambient-temperature data collection at SPring-8

Recent advancements in ambient-temperature crystallography, including time-resolved studies, have revealed the dynamic properties of proteins and renewed interest in room-temperature experiments (Fraser *et al.*, 2011 ▸). However, the fragile and hydrated nature of protein crystals makes them susceptible to dehydration and temperature fluctuations, posing challenges for ambient-temperature data collection.

**Figure 10:**
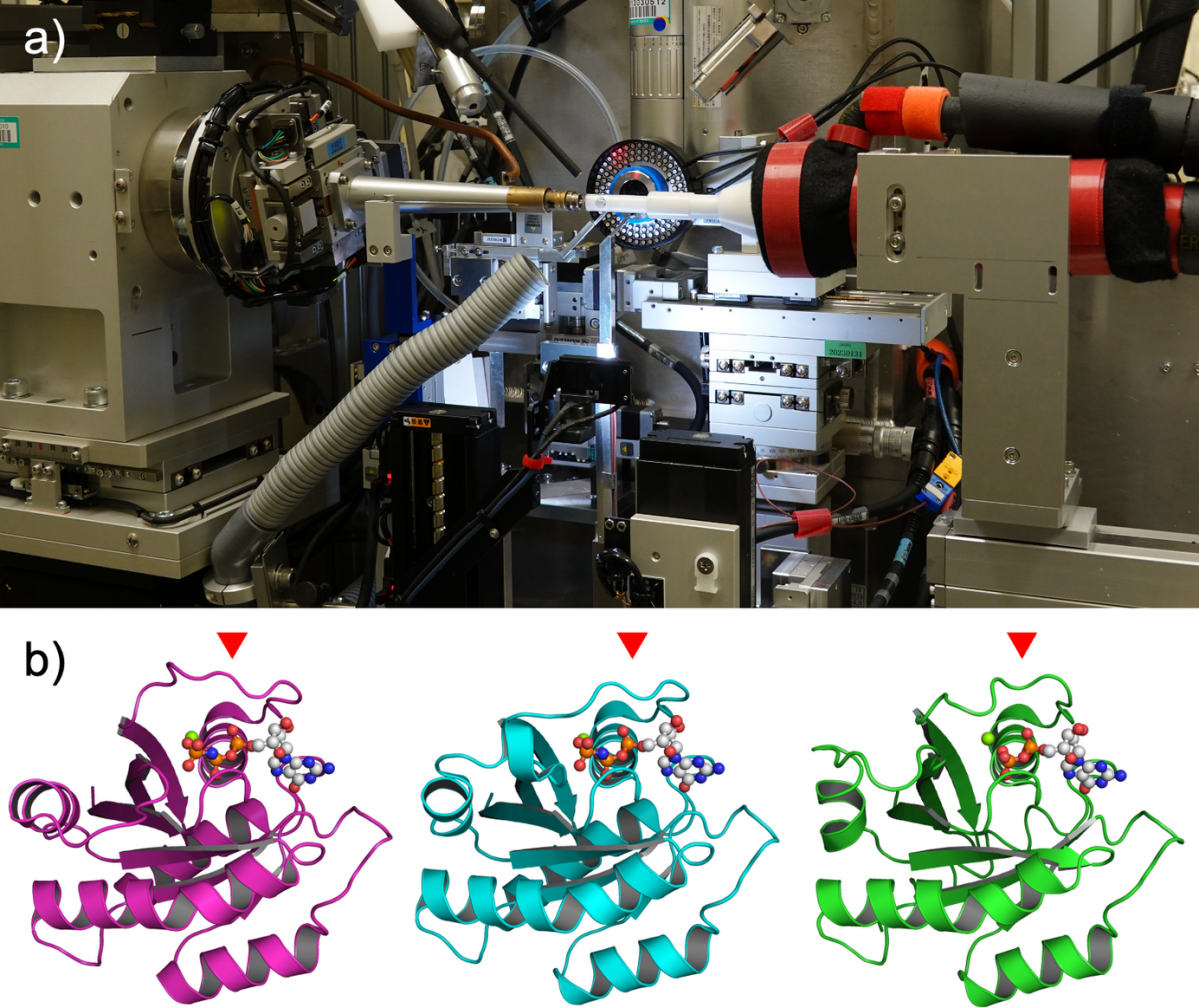
Ambient-temperature measurement setup and its result. (*a*) A humidifier setup at SPring-8 BL41XU. (*b*) Three structural states of H-Ras, which regulates cell signalling. The switch I loop, indicated with red triangles, shows structural polymorphism corresponding to bound nucleotide, GTP bound open (state 1, magenta), GTP bound close (state 2, cyan) and GDP bound forms (green).

Traditional techniques such as glass capillaries and humidifiers have been refined to overcome these issues. A notable improvement is the ‘humid air and glue coating (HAG)’ method, which incorporates hydro­philic polymer glue for crystal coating (Baba *et al.*, 2013 ▸). This method eliminates the need for permeable cryoprotectants, such as glycerol, allowing consistent measurements under cryogenic and room-temperature conditions [Fig. 10 ▸(*a*)]. Its capillary-free design also facilitates external modulation of the sample environment, making it highly suitable for structural polymorphism and time-resolved analyses.

A groundbreaking application of the HAG method involved Ras, a proto-oncogene product and small G-protein, and a longstanding target in drug discovery. Previously, only the closed conformation of its active state (without a druggable binding pocket) had been resolved. By inducing pH changes through humidity conditioning, which was caused by the volatilization of acetic acid contained in the mother liquor due to continuous blasts of humid air, we captured the inactive open conformation of Ras (Matsumoto *et al.*, 2016 ▸), a structure previously hypothesized via NMR studies [Fig. 10 ▸(*b*)]. This discovery opens new avenues for designing Ras-targeted drugs.

This method has also been expanded to studies across the low- and high-temperature ranges (Baba *et al.*, 2019 ▸). Notable applications include the analysis of copper-containing amine oxidase, a key enzyme in primary amine metabolism. By manipulating pH and temperature, researchers observed conformational changes in its topa­quinone cofactor, shedding light on its reaction mechanisms (Murakawa *et al.*, 2019 ▸). The low-temperature HAG method has also been utilized in SF-ROX experiments on cytochrome c oxidase (CcO), as described above, further demonstrating its versatility in studying protein conformational dynamics. Applications at higher temperatures are now under investigation. Jacobs *et al.* (2024 ▸) introduced body-temperature protein crystallography, highlighting its potential to resolve physiological structures. This breakthrough could pave the way for future innovations in drug discovery.

These advances set the stage for further integrating time-resolved crystallography to explore protein functionality and dynamics. By bridging static structural snapshots and dynamic analyses, ambient-temperature experiments at SPring-8 provide critical insights into the molecular mechanisms underlying protein activity.

## 5. Future direction of structural biology at SPring-8

SR has revolutionized MX, driving significant advancements in measurement accuracy and the development of novel phase determination methods. The high-brilliance microbeam technology at SPring-8 has expanded the scope of structural analysis to include highly challenging targets, such as membrane proteins and protein complexes – key areas for life science research and drug discovery. These developments are expected to strengthen foundational technologies for structure-based drug design (SBDD), facilitating breakthroughs in vaccine development and treatments for emerging infectious diseases, including new therapeutics for the coronavirus.

Structural dynamics analysis that directly explores protein function mechanisms will be critical for advancing MX. The field is poised for transformation with the advent of next-generation SR facilities. MAX-IV in Sweden (Tavares *et al.*, 2014 ▸; Robert *et al.*, 2023 ▸) became the world’s first ultra-low-emittance, fourth-generation SR facility in 2016, offering ultra-brilliance and highly coherent SR. Similarly, the ESRF completed its upgrade to ESRF-EBS in 2020 (Raimondi *et al.*, 2023 ▸), with additional upgrades planned for APS and SLS in Switzerland. At SPring-8, the ‘SPring-8-II project’ (Tanaka *et al.*, 2024 ▸) is underway, promising X-ray beams 100 times brighter than the current facility. Scheduled for operation in 2029, this next-generation SR facility will enable innovations such as high-resolution structural analysis from even smaller crystals, faster time-resolved measurements and deeper insights into structural dynamics. Moreover, it is anticipated that the serial crystallography method at SR facilities will improve substantially, facilitating seamless integration with XFEL facilities. While current SR serial methods face limitations in time resolution and crystal size, this synergy can revolutionize protein dynamics research and enable groundbreaking discoveries in structural biology.

Meanwhile, cryo-electron microscopy (cryo-EM) has experienced transformative advances, particularly in single-particle analysis, and has been recognized with the 2017 Nobel Prize in Chemistry (The Nobel Prize in Chemistry, 2017 ▸). The integration of cryo-EM and crystal structure analysis is ushering in an era of unprecedented visualization of protein structures. At SPring-8, a high-end cryo-EM facility has been established for public use, laying the groundwork for complementary and integrated applications with SR.

Machine learning is also reshaping structural biology, with tools like *AlphaFold* (Abramson *et al.*, 2024 ▸) offering highly accurate structure predictions. The success of *AlphaFold*, recognized with the 2024 Nobel Prize in Chemistry (The Nobel Prize in Chemistry, 2024 ▸), marks a turning point in studying protein function in biochemical and structural biology. SPring-8 is poised to play a pivotal role in this evolving landscape by contributing experimentally determined, high-precision structures to enhance structure prediction databases. Moreover, the facility advances rapid phase determination methods that leverage predicted structures.

Digital transformation (DX) is central to these advancements, enabling streamlined workflows from measurement preparation to data analysis. The ongoing development of cutting-edge measurement techniques and software at SPring-8 ensures efficient structural analysis of previously intractable samples. These innovations will not only expand the boundaries of protein structural analysis but also drive progress in understanding biological mechanisms and facilitating the design of novel therapeutics.

In conclusion, SPring-8 is committed to advancing structural biology by integrating state-of-the-art technologies, fostering complementary approaches and pushing the frontiers of structural analysis. Through its contributions, SPring-8 will continue to play a vital role in shaping the future of life sciences and biomedicine.
